# Structured team-oriented program to follow patients after vena cava filter placement: a step forward in improving quality for filter retrieval

**DOI:** 10.1038/s41598-021-82767-3

**Published:** 2021-02-10

**Authors:** Salah D. Qanadli, Kiara Rezaei-Kalantari, Laurence Crivelli, Francesco Doenz, Anne-Marie Jouannic, David C. Rotzinger

**Affiliations:** 1 Cardiothoracic and Vascular Division, Department of Diagnostic and Interventional Radiology, Lausanne University Hospital and University of Lausanne, Rue du Bugnon 46, 1011 Lausanne, Switzerland; 2 Rajaie Cardiovascular Medical and Research Center, Iran University of Medical Sciences, Tehran, Iran

**Keywords:** Vascular diseases, Thromboembolism, Thrombosis, Medical research, Outcomes research

## Abstract

To reduce inferior vena cava filter (IVCF) related complications, retrieval is recommended whenever possible. Nevertheless, IVCF retrieval rates remain lower than expected, likely due to insufficient follow-up after placement. We evaluated the value of a structured program designed to follow patients by the interventional radiology team up to 5 months after IVCF placement. We prospectively enrolled 366 consecutive patients (mean age 64 ± 17 years; 201 men and 165 women) who benefited from IVCF between March 2015 and February 2020. The program consisted of advising the patient and clinicians to consider IVCF retrieval as soon as possible (standard workflow) and systematically planning an additional follow-up visit at 5-month. Clinical and technical eligibility, as well as technical success for retrieval (TSR) were evaluated. At 5-months, 38 (10.4%) patients were lost to follow-up, and 47 (12.8%) had died. Among survivors, the overall retrieval rate was 58%. The retrieval rates were 83% and 97% for the clinically eligible and technically eligible patients for retrieval, respectively. The 5-month visit enabled 89 additional retrievals (47.8%) compared to the standard workflow. No significant difference was seen in TSR before and after 5 months (p = 0.95). Improved patient tracking with a dedicated IVCF program results in an effective process to identify suitable patients for retrieval and drastically improves retrieval rates in eligible patients. Involving interventionalists in the process improved IVCF patient management.

## Introduction

Inferior vena cava filters (IVCFs) have been widely used since the 1970s to prevent pulmonary embolism (PE) in patients with or without venous thromboembolic disease. Reportedly, up to 17% of patients with PE receive an IVCF^1^. Despite their frequent use, the indications for, clinical benefits, and long-term outcomes of IVCF use are regularly a matter of debate and concern, resulting in non-standardized guidelines and sometimes conflicting recommendations^2^.

Recent systematic reviews and meta-analyses^3,4^ have shown that patients with an IVCF have a lower risk of recurrent PE, an increased risk of deep venous thrombosis (DVT), trend of lower PE-related mortality, and no change in short-term all-cause mortality. A recent study conducted in patients with cancer-associated venous thromboembolism who received an IVCF confirmed the absence of significantly different all-cause mortality, but showed a lower PE-related mortality^5^. DVT is the most common complication, occurring in up to 20% of patients^6^, even after early initiation of anticoagulation in patients with IVCFs^7^. Moreover, IVCF use is associated with multiple mechanical complications, mostly with long-term use^3^. These limitations justified the promotion of temporary filtration and retrieval of the filtration device as soon as possible. Optional IVCFs, which can be used either as retrievable or permanent filters, have been developed to overcome these limitations^8^.

Despite the spread of optional IVCF use and recommendations for retrieval, low rates of effective filter retrieval have been observed. In recent literature, the IVCF retrieval rate ranges from 1 to 64%^3,9–13^, with a mean of 34%^3^. The main reason is a lack of follow-up after filter placement and failure to inform patients regarding the benefits of retrieving the filter if possible^3,14^.

Given the risk of adverse events associated with a long IVCF dwell time, retrieval plans and structured follow-up strategies are being established in many hospitals^14–17^. Such approaches should allow definition of the conditions in which an optional filter can be retrieved and ensure that all filters eligible for retrieval are effectively retrieved. Several follow-up strategies have been proposed, and the key to successful follow-up and filter removal is the presence of one central actor, being the operator or his team. The operator is responsible for the IVCF implantation procedure, but also the follow-up after insertion^18,19^. In other words, the operator’s team is in charge of informing both the patient and the referring clinician about the possibility and timing of retrieval, and to ensure that no filter is left in place without re-evaluating the situation. Following this rationale, we implemented a quality improvement program in the Department of Radiology to improve our practice.

The objective of the current study was to report our structured program for patient follow-up after IVCF placement and evaluate its impact on IVCF retrieval rate.

## Materials and methods

The Cantonal Commission on Ethics in Human Research (CER-VD) approved this prospective study protocol. All procedures conducted by the Lausanne University Hospital under the Federal Act on Research involving Human Beings (Human Research Act, HRA). Informed consent was obtained from all participants.

### Study design

The study was designed to prospectively include all patients referred to the Department of Radiology for IVCF placement between March 2015 and February 2020. At our institution, all IVCF placement procedures are performed in the Department of Radiology. Since 2015, all patients have received the same optional IVCF (Denali; Bard Peripheral Vascular, Inc., Tempe) when indicated.

Inclusion criteria were age over 18 years, an indication for IVCF placement, and consent to be enrolled. Patients with a contraindication for placement of a Denali filter per the instructions for use (i.e., stenosis or compression of the inferior vena cava or an anatomic variant of the inferior vena cava system) and patients receiving peri-procedural pharmaco-mechanical thrombectomy for PE or DVT were excluded from the study.

Indications for IVCF placement are defined by the Standards of Practice of the Society of Interventional Radiology and separated into therapeutic (i.e., a thromboembolic event was documented) and prophylactic categories^18^. By local multidisciplinary consensus, prophylactic indications are limited due to controversies about the benefit-risk balance^20^*.*

The standard workflow at our institution incorporates a number of steps to maximize patient and clinician awareness regarding the need to remove the filter once it is not needed anymore:

1. Following IVCF placement and before discharge, the instructions for filter retrieval were documented as soon as possible in the medical report.
2. A copy of the medical report was sent to the patient’s primary care physician, including instructions encouraging a clinical visit with the IVCF IR referent whenever the filter was no longer needed.
3. The patient was informed of the strategy after IVCF placement.

**Figure 1:**
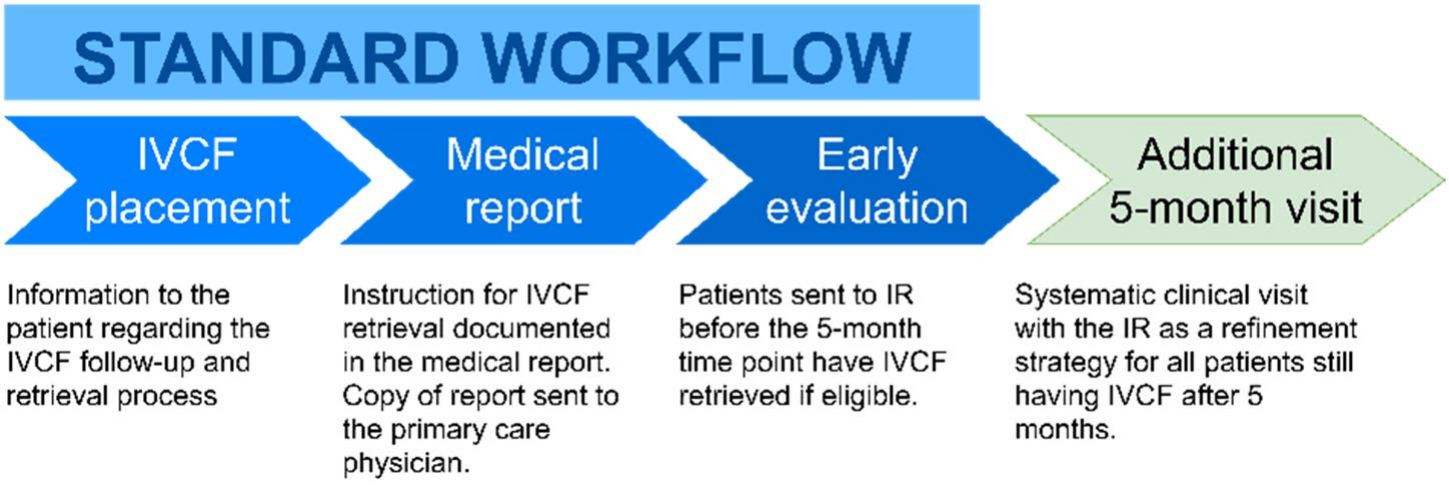
Prospective longitudinal study design. *IVCF* inferior vena cava filter, *IR* interventional radiologist.

Beyond that, the study consisted of adding a structured follow-up program as a refinement strategy to ensure that all subjects receive an assessment for potential filter retrieval (Fig. 1). To this end, we scheduled a systematic follow-up visit at 5 months; if the filter was removed early (before 5 months), the follow-up visit was cancelled. Clinical visits for follow-up were conducted centrally by a single member of the interventional radiology (IR) team (*blinded*) assigned to this task (IVCF referent). To balance the risks of irretrievability due to prolonged dwell time and simultaneously avoid redundant patient visits, the time to re-evaluation was set at 5 months.

When assessing the impact of the 5-month follow-up visit on filter retrieval, patients who had their IVCF removed early were considered to be part of the standard workflow, and those who completed the 5-month follow-up visit were considered as being part of the standard workflow + additional follow-up visit since they could potentially benefit from the refinement strategy. By design, any filter removal procedures performed after 5 months reflect the impact of the additional follow-up visit provided by IRs on the IVCF retrieval rate.

### Filter placement procedure

Placement was performed using the standard technique from femoral, jugular or subclavian venous access under local anesthesia. First, phlebography of the inferior vena cava was performed to locate the renal veins and estimate the diameter of the vena cava. The filter was then deployed under fluoroscopic guidance. The intended optimal position is immediately infra-renal, below the most caudal renal vein. In the case of an aberrant retro-aortic renal vein, the filter was placed below the most caudal right renal vein. If the filter was tilted (primary tilt) or malpositioned (primary malposition), no additional maneuvers were recommended, so the tilt or malposition was noted in the report.

### Filter removal procedure

Criteria for filter removal included the clinical status and technical retrievability of the filter as described in the outcome section. The procedure was planned on a day case basis and performed percutaneously by jugular venous access under local anesthesia. The filter was withdrawn under fluoroscopic guidance using a dedicated snare system (Bard). In the case of snaring failure, more advanced techniques could be used at the radiologist’s discretion.

### Outcome assessment

The primary endpoint was the retrieval rate in the standard workflow and standard workflow with additional 5-month follow-up visit, in eligible patients. Secondary endpoints were mechanical issues that compromise filter retrieval and complications during the retrieval procedure. Mechanical issues were categorized as filter tilt, penetration, migration or material embolization, fracture, and vena cava stenosis/occlusion, as previously reported in the Standards of Practice of the Society of Interventional Radiology^18^. Technical success for IVCF placement corresponded to successful optimal placement.

Clinical criteria for permanent filtration were based on life expectancy (< 6 months), age (> 90 years), anticoagulation failure after re-introduction, long-term contraindication of anticoagulation, and long-term immobilization. If, on the day of placement, the patient presented with at least one clinical criterion, the filter was declared permanent, and the 5-month visit canceled. Patients with no criteria for permanent filtration were considered clinically eligible for filter retrieval.

Technical filter retrievability assessment was performed with indirect CT venography, routinely obtained before evaluating patients for filter retrieval^21–23^. Technical irretrievability was based on the presence of a large thrombus in the filter (> 30% of filter volume) or deep penetration. Filter tilt was not considered a criterion of irretrievability. Patients with no irretrievability criteria were considered technically eligible for retrieval. Patients who had a thrombus in the filter were treated with anticoagulant therapy and reassessed for possible retrieval. Technical success was defined as successful retrieval of the filter.

Procedural complications were registered and defined as minor (i.e., no consequences requiring hospitalization > 23 h post-procedure) or major (i.e., supplementary stay from 1 day to a fatal event)^18^. Deaths during the 5 months following IVCF placement were documented.

### Statistical analysis

Statistical analysis was conducted using R 3.5.3 (R Core Team 2015). Continuous variables were expressed as mean ± standard deviation. Categorical variables were presented as absolute numbers and proportions and compared using the chi-squared or Fisher exact test as appropriate. Percentages were used to report the occurrence rate in each category. p < 0.05 were considered significant. Patients lost to follow-up or who had died were excluded from the analysis.

## Results

### Patient characteristics and IVCF placement

A total of 366 consecutive patients (201 men, 54.9%; 165 women, 45.1%) were enrolled in the study. The mean age was 64 ± 17 years. The majority of patients had an IVCF placed for an absolute indication and simultaneous contra-indication for anticoagulation (62.3%), followed by complications of anticoagulation therapy (20.6%). The filters were also placed for relative indications of surgery planned for patients with thromboembolic disease (4.7%), inability to achieve or maintain a satisfactory anticoagulation level (2.4%), an iliocaval deep vein thrombosis (1.8%), or a recurrent deep vein thrombosis (1.2%). Prophylactic indications consisted of high risk for thromboembolic disease due to risk factors (4.1%) and severe trauma (2.9%; Table 1).

**Table 1:** Demographic characteristics and indications of filter placement in the study population (survivors with 5 month follow-up).

	Standard workflow	Standard workflow + follow-up	Total
Patients (n)	150	131	281
Age (years ± SD)	64 ± 18	63 ± 17	64 ± 17
Sex (M/W)	85/64	74/57	201/165
**IVCF indication**			
Absolute			
PE and contraindication to ACT			147
Proximal DVT and contraindication to ACT			56
PE/DVT and complication of ACT			26
Relative			
Recurrent PE under ACT			12
Recurrent DVT			6
Inability to achieve adequate anticoagulation			8
DVT and severe trauma			11
Prophylactic			
High risk patients			15

*M* men, *W* women, *IVCF* inferior vena cava filter, *ACT* anticoagulation therapy, *PE* pulmonary embolism, *DVT* deep venous thrombosis.

A total of 370 IVCFs were placed in 366 patients; 359 (97%) were placed in the infra-renal position. Four patients received a second filter placed several months (mean 862 ± 1176 days) after the first filter retrieval. All procedures for IVCF placement were successful, and no periprocedural complication was observed.

### Follow-up and filter retrieval

At 5 months, 38 (10.4%) patients were lost to follow-up, and 47 had died (12.8%). Thirty-two of the deaths (68%) occurred within 30 days. Thus, 281 patients were analyzed regarding IVCF retrieval.

**Figure 2:**
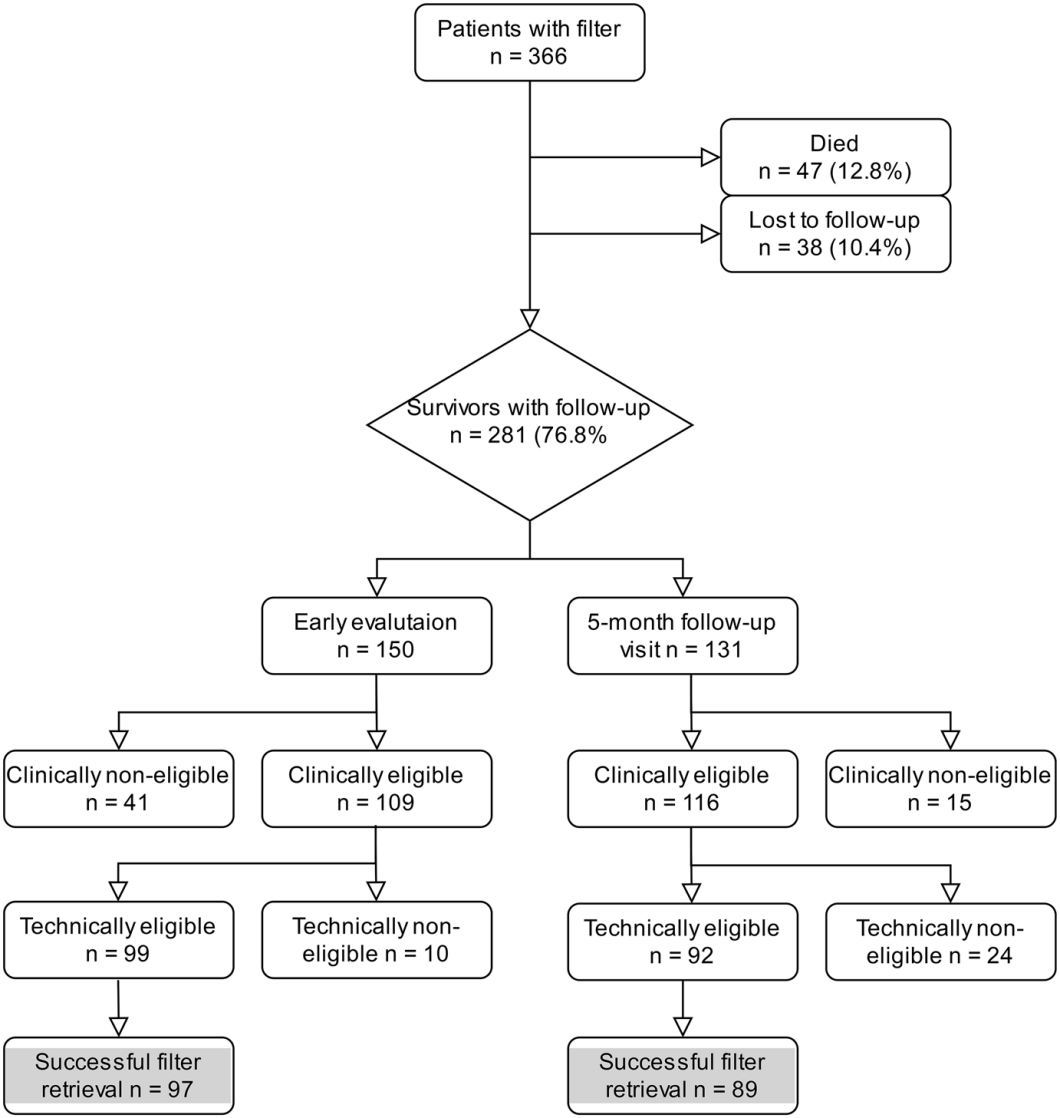
Flowchart. Technically eligible patients (n = 191) include 6 patients initially deemed technically non-eligible due to thrombus who received anticoagulant therapy and achieved technical eligibility.

A total of 150/281 (53.3%) patients benefited from early evaluation with retrieval (mean 48 ± 36 days), including 105 before discharge (70%) and 45 (30%) during an early visit with the IVCF IR referent. The 5-month follow-up strategy allowed to evaluate 131 (46.6%) more patients for filter retrieval. In 10 patients (3.5%), the IVCF was declared permanent at the time of placement. Among the clinically eligible patients, 12 (5.3%) refused the retrieval procedure. Overall, a total of 225/281 (80%) patients were clinically eligible for IVCF retrieval. In the standard workflow, 109/150 (72.6%) patients were found to be clinically eligible and 99/109 (90.8%) technically eligible for retrieval, whereas 116/131 (88.5%) patients who had 5-month follow-up were found clinically and 92/116 (79.3%) technically eligible, p < 0.001 and 0.02, respectively. Reasons for technical ineligibility are detailed in Table 2. A process flowchart (Fig. 2) from patient enrollment to outcome is provided for clarity.

**Table 2:** Technical eligibility for IVCF retrieval in clinically eligible patients.

	Standard workflow	Standard workflow + 5-month follow-up	Total
Age (years)	79.6	65.5	
Eligible	99/109 (90.8%)	92/116 (79.3%)	191/225 (84.9%)
Non-eligible	10/109 (9.2%)	24/116 (20.6%)	34/225 (15.1%)
**Findings in technically non eligible patients**			
Thrombus^a^	5/10 (50%)	8/24 (33.3%)	13/34 (38.2%)
Penetration^b^	1/10 (10%)	9 (37.5%)	10/34 (29.4%)
Tilt	4/10 (40%)	7/24 (29.2%)	7/34 (20.6%)

*IVCF* inferior vena cava filter.

^a^Volume thrombus within the filter more than 30%.

^b^Patients with transmural deep penetration.

Technically successful retrieval was achieved in 186/191 (97.4%) patients. Among patients initially considered technically non-eligible, 13/225 (5.8%) had a thrombus inside the filter, 5 patients in the standard workflow, and 8 patients with 5-month follow-up. After anticoagulation therapy, six additional patients were re-categorized as eligible for retrieval, which was performed successfully in five of them. The overall technical success was not significantly different in the standard workflow (98%, 97/99) and in filters removed after the follow-up visit (96.7%, 89/92; p = 0.95). The complications leading technical ineligibility (thrombus and penetration) and failure (penetration or tilt) are reported in Table 3. All failed retrieval procedures were associated with at least one mechanical complication.

**Table 3:** Retrieval rates achieved at different stages of the study.

Patients	n	Filter retrieval rate (No.)
Overall population	366	51% (186)
Survivors	319	58% (186)
Survivors with 5-month follow-up	281	66% (186)
**Standard workflow**		
All clinically eligible*	225	43% (97)
All technically eligible§	191	51% (97)
**Standard workflow with 5-month visit**		
Clinically eligible*	225	83% (186)
Technically eligible§	191	97% (186)

*p value < 0.001.

^§^p value < 0.001.

**Figure 3:**
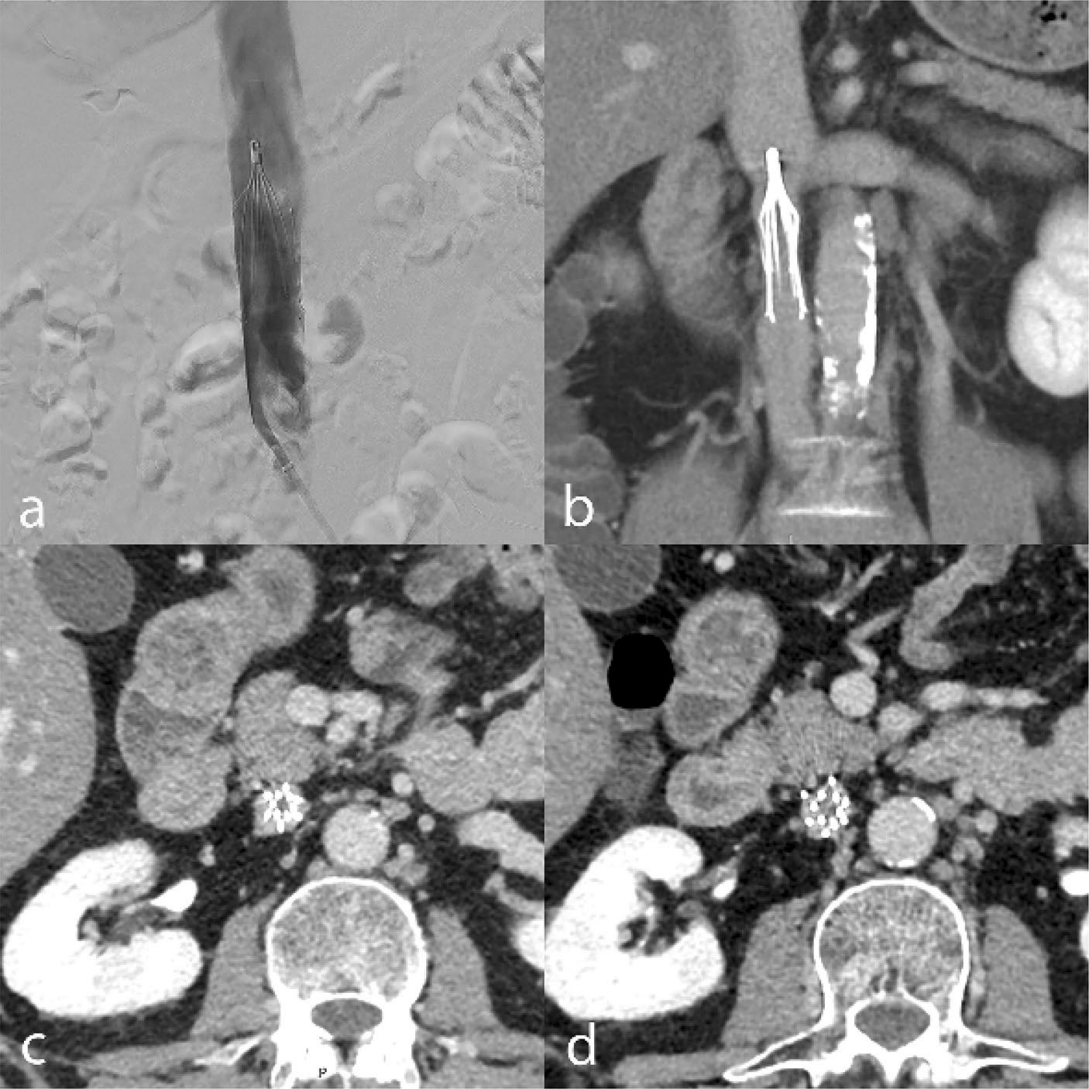
Post-deployment subtracted cavography shows IVCF positioned in infrarenal position and absence of IVC stenosis, in a patient with femoral and iliac DVT under ACT (**a**); CT venography obtained 5 months later shows IVC stenosis on coronal reformatted image (**b**), and thrombus trapped inside the IVCF (**c**,**d**), leading to technical ineligibility for retrieval. *ACT* anticoagulation therapy, *CT* computed tomography, *DVT* deep venous thrombosis, *IVC* inferior vena cava, *IVCF* inferior vena cava filter.

**Figure 4:**
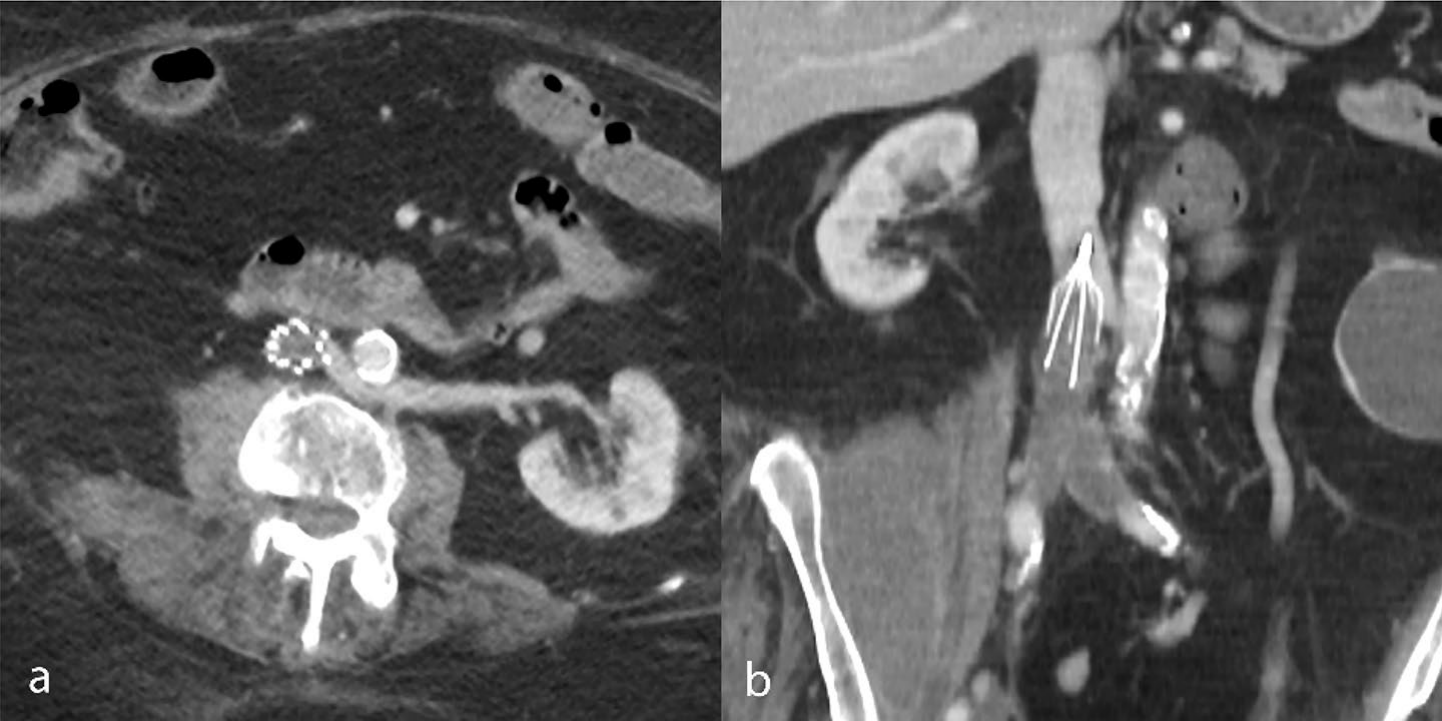
CT venography obtained one month after IVCF placement shows filter tilt and acute DVT of the iliac veins and infrarenal IVC on axial (**a**) and coronal reformatted image (**b**), leading to technical ineligibility for retrieval. *CT* computed tomography, *DVT* deep venous thrombosis, *IVC* inferior vena cava, *IVCF* inferior vena cava filter.

**Figure 5:**
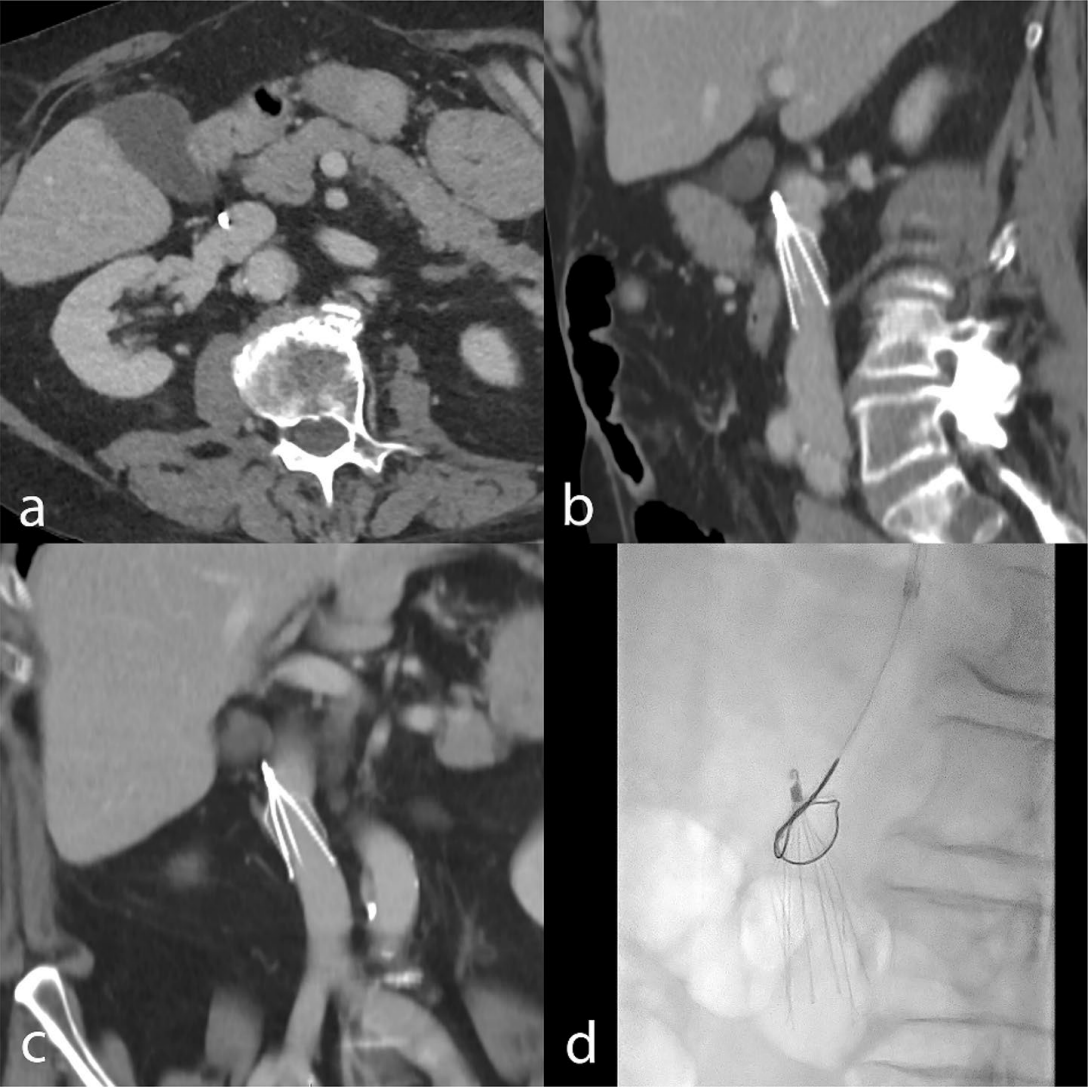
In a clinically eligible patient for IVCF retrieval, CT venography showed an embedded filter hook (**a**) due to a tilt visible both on sagittal (**b**) and coronal (**c**) reformatted images. Fluoroscopy image during the procedure shows unsuccessful attempt to retrieve the IVCF using a single-loop snare system (**d**). *CT* computed tomography, *IVCF* inferior vena cava filter.

The overall retrieval rate was 50.8% (186/366). Among survivors, the overall retrieval rate was 66.2% (186/281). The retrieval rates at the end of the study were 82.7% (186/225) and 97.4% (186/191) for the clinically eligible and technically eligible patient subsets, respectively. The dwell time before retrieval was 39.7 days in the standard workflow and 218.7 days in standard workflow with 5-month visit. The 5-month visit enabled retrieval in 89 additional patients (47.8%, 89/186) compared to the standard workflow, leading to a significantly higher overall retrieval rate Table 3. Figures 3, 4, and 5 show examples of patients seen at the 5-month follow-up visit who were clinically eligible but either technically non-eligible (Figs. 3 and 4), or technically eligible with retrieval failure (Fig. 5).

## Discussion

The rationale for temporary filtration is based on well-founded concerns regarding long-term complications of permanent IVCFs, particularly in young patients with a temporary contraindication for anticoagulation. This has led to the development of retrievable filters. Though the underlying purpose of retrieving unnecessary IVCFs is relevant, strategies for identifying patients suitable for IVCF removal still lack in broader practice, resulting in limited clinical impact. Therefore, the benefit of placing retrievable filters is questionable, and improvements in the process have to be considered.

Several studies investigating factors associated with low IVCF retrieval rates have pointed out poor patient follow-up as the primary reason^10^. Utilization of IVCFs and the subsequent retrieval rates vary widely by geography and institution, suggesting that appropriate tracking of patients and specific recommendations for adequate follow-up would likely improve patient safety. Our study demonstrates that, from a clinical point of view, it is not reasonable to aim for retrieving IVCFs in all patients. For multiple reasons, the overall retrieval rate was ~ 51% in our study. However, a structured follow-up with the systematic 5-month visit concept provided a very high rate of retrievability, reaching 99% in eligible patients. The addition of a 5-month visit significantly improved the retrieval rate, as roughly half (48%) of the retrieval procedures were performed after the 5-month visit. These results are comparable or slightly better than those reported in the literature, mainly in retrospective reviews or comparisons. In the PREPIC2 study^24^, a randomized multicenter trial for retrievable IVCFs, more than 90% of IVCFs were removed because of a 3-month follow-up visit. Another study comparing retrieval rates for 100 temporary filters with and without IVCF clinic reported a substantial improvement, from 29% without to 60% with an IVCF clinic^25^.

The time to a re-evaluation of IVCF retrievability may be a matter of discussion. The risk of non-retrievability increases with prolonged dwell time^20,26^ and depends on the indication for placement. For example, a recent study showed that trauma patients have a retrieval rate twice as high as other patients^27^ due to retrieval before discharge. In addition, complication rates have been reported to be higher 6 months after placement^28^, discouraging lengthy follow-up delays. As the main indications at our institution were non-prophylactic, we designed the follow-up visit for 5 months to balance between benefits and risks, as it is far enough away from the placement to avoid recalling every patient and giving approximately a month to plan and complete the retrieval procedure within 6 months. Our strategy of selecting 5 months seems appropriate regarding the technical success of retrieval, as no significant difference was found between early retrieval and retrieval after 5 months. Directly combining the scheduling of the 5-month visit with the IVCF placement report is a simple and effective means of following the patient and improving the quality control of the service provided. However, it is critical to inform the patient about the follow-up visit the day of placement to increase comprehension of the filter management process and decrease patient apprehension regarding further investigations. Centralizing all visits with the IVCF IR referent smoothes out the process and homogenizes patient management.

Interestingly, the rates of eligible patients for filter retrieval were similar in both patient subsets, which can be attributed to a systematic and thorough assessment of eligibility criteria by the IVCF IR referent for identifying suitable patients. Other alternatives have been reported. A multidisciplinary team^29^ or a dedicated IVCF clinic^25^ are effective approaches that have shown promising results. However, they are more complex to implement.

Controversies regarding the use of retrievable IVCFs for each patient are justified due to a higher risk of complications than permanent filters when left in place^20,29–31^. Nevertheless, the strategy of offering retrieval appeared to be more appropriate, as only 3.5% of the delivered filters were declared permanent at the time of placement. Deciding on permanent filtration at the time of deployment is problematic, as the majority of IVCFs are being placed in an emergency setting. In the present study, despite the effectiveness of a prospective follow-up program, 33% of filters were left in place, and 5.8% were unsuccessfully retrieved. Therefore, considering the long-term complication rates of retrievable filters, it is critical to select the appropriate filter for insertion. The MAUD database, in which IVCF-related adverse events are reported, clearly indicates that all filters are not equal^21^. However, asymptomatic patients with unsuccessful IVCF retrieval and significant mechanical disorders (i.e., deep penetration or a tilt of more than 15°) rarely develop clinical complications^30^.

One of the major factors associated with irretrievability of the filter was death before removal (13.1%). This is consistent with a recent systematic review analyzing reasons for non-retrieval^31^, in which the mortality was as high as 19.4%. In the same report, patient refusal was the reason for 2.4% of non-retrievals. In our study, patient refusal occurred in 5.3% of eligible patients, which represents 3.3% of the whole study population.

The present study has several limitations. Despite its prospective design, 10% of patients were lost to follow-up. This is similar to the percentage (13%) recently reported by a systematic review on the utility of IVCF^31^. Our institution is a tertiary center, and several patients were transferred from other centers for filter placement, making it challenging to follow them after the procedure. Furthermore, the study did not integrate a cost-effectiveness analysis that may help us understand such follow-up programs' financial impact. Finally, as designed, this study did not allow evaluation of long-term prognosis and the predictive value of demographic characteristics, filter dwell-time, and serological biomarkers for VTE recurrence and death. Conducting additional mid-term and long-term follow-up in a larger cohort is a relevant future perspective, intending to identify patients at increased risk of recurrent VTE.

In conclusion, improved patient tracking with a dedicated IVCF program results in an effective process for identifying suitable patients for retrieval and drastically improves retrieval rates in eligible patients. By implementing well-organized and straightforward follow-up programs, the mission to avoid any potential long-term complications of these temporary devices and improve patient care may be fulfilled.
